# Detection of multiple mutations in urinary exfoliated cells from male bladder cancer patients at diagnosis and during follow-up

**DOI:** 10.18632/oncotarget.11883

**Published:** 2016-09-07

**Authors:** Rossana Critelli, Francesca Fasanelli, Marco Oderda, Silvia Polidoro, Manuela Bianca Assumma, Clara Viberti, Mirko Preto, Paolo Gontero, Giuseppina Cucchiarale, Irene Lurkin, Ellen C. Zwarthoff, Paolo Vineis, Carlotta Sacerdote, Giuseppe Matullo, Alessio Naccarati

**Affiliations:** ^1^ Molecular and Genetic Epidemiology Unit, Human Genetics Foundation, Turin, Italy; ^2^ Department of Medical Sciences, University of Turin, Turin, Italy; ^3^ Department of Surgical Sciences, Urology, University of Turin, Turin, Italy; ^4^ Genomic Variation in Human Populations and Complex Diseases Unit, Human Genetics Foundation, Turin, Italy; ^5^ Department of Urology, Humanitas Cellini, Turin, Italy; ^6^ Department of Pathology, Erasmus MC, Rotterdam, The Netherlands; ^7^ Department of Surgery and Cancer, Imperial College London, London, UK; ^8^ Unit of Cancer Epidemiology, Centre for Cancer Prevention (CPO-Piemonte), Turin, Italy

**Keywords:** bladder cancer, urine mutation analyses, TERT, recurrence

## Abstract

Most bladder cancer (BC) patients need life-long, invasive and expensive monitoring and treatment, making it a serious burden on the health system. Thus, there is a pressing need for an accurate test to assist diagnosis and surveillance of BC as an alternative to cystoscopy. Mutations in human *TERT*, *FGFR3*, *PIK3CA,* and *RAS* genes have been proposed as potential molecular markers in bladder tumor. Their concomitant presence in urine samples has not been fully explored.

We investigated a panel of mutations in DNA from exfoliated urinary cells of 255 BC patients at diagnosis. Forty-one mutations in *TERT, FGFR3, PIK3CA,* and *RAS* were analyzed by SNaPshot assay in relation to clinical outcome. In 81 of these patients under surveillance, the same set of mutations was screened in additional 324 samples prospectively collected.

The most common mutations detected in urine at diagnosis were in the *TERT promoter.* In non-invasive BC, these mutations were related to high risk and grade (p<0.0001) as well as progression to muscle-invasive disease (p=0.01), whereas *FGFR3* mutations were observed in low-grade BC (p=0.02) and patients with recurrences (p=0.05). Stronger associations were observed for combined *TERT* and *FGFR3* mutations and number of recurrences (OR: 4.54 95% CI: 1.23-16.79, p=0.02). Analyses of the area under the curve for combinations of mutations detected at diagnosis and follow-up showed an accuracy of prediction of recurrence of 0.80 (95% CI: 0.71-0.89).

Mutations in urine of BC patients may represent reliable biomarkers. In particular, *TERT* and *FGFR3* mutations have a good accuracy of recurrence prediction.

## INTRODUCTION

Bladder cancer (BC) is the seventh most frequently diagnosed malignancy worldwide in men [1] and the most common cancer of the urinary tract, with urothelial carcinoma being the dominant histology in more than 90% of BC cases [2]. Approximately 75% of BC patients present with non-muscle-invasive BC (NMIBC), of which 70% are confined to the mucosa (Ta), 20% to the submucosa (T1), and 10% are carcinoma *in situ* (CIS) [3]. According to current guidelines, these lesions are treated with transurethral resection of the bladder (TURB) followed by one intravesical instillation with mitomycin C. Patients with intermediate or high risk of progression to muscle-invasive cancer (MIBC) subsequently undergo several cycles of Bacillus Calmette-Guerin (BCG) instillations according to the guidelines of the European Association of Urology [4]. The remaining cases are MIBC for which the standard of care is radical cystectomy with pelvic lymph node dissection, with possible neoadjuvant or adjuvant chemotherapy [5].

MIBC cases exhibit a poor prognosis with a high risk of post-operative complications and an estimated five-year overall survival (OS) rate of 66% [5]. Although survival for NMIBC cases is over 90%, patients with these tumors are subject to high rates of recurrence (up to 78% at five years) and progression to muscle-invasive (up to 45% at five years) despite treatment [4]. For these patients, the goal is to detect and treat recurrences early in order to avoid the progression to MIBC. Currently, the only approach is a lifelong surveillance consisting of cystoscopy with or without biopsies and voided urine cytology [4, 5]. To improve the management of BC and at the same time decrease the costs, it is essential to find a non-invasive, highly sensitive, and specific molecular biomarker that allows detection of recurrences in urine.

BCs are derived from the urothelium involving, at least, two different genetic pathways and distinct progenitor cell types [6]. NMIBCs are characterized by frequent mutations in the fibroblast growth factor receptor 3 (*FGFR3*) and three of the five human *RAS* genes such as Harvey rat sarcoma viral oncogene homolog (*HRAS*), Kirsten rat sarcoma viral oncogene homolog (*KRAS*), and neuroblastoma RAS viral (v-ras) oncogene homolog (*NRAS*) [7–9]. Also, a large proportion of human bladder tumors show alterations in some of the phosphatidylinositol 3-kinase (*PI3K*) pathway components; in particular, the phosphatidylinositol 3-kinase catalytic subunit alpha (*PIK3CA*) gene [10] that is involved in controlling cell growth, survival, and proliferation and has been considered a promising target for cancer therapy [11, 12]. MIBC cases, which show a propensity to progress to local and distant metastasis, often contain defects in tumor suppressor genes [13–15]. The telomerase reverse transcriptase (TERT) promoter, an important element of telomerase expression, has emerged as a target of cancer-specific mutations. Originally described in melanoma, the mutations in human *TERT* promoter, mainly at -124 and -146 base pair positions from the ATG start site, have been shown to be common in certain tumor types including BC of all stages with mutation frequencies of up to 80% [16–20].

The choice of a urine-based assay to analyze BC genetic profiles poses a major advantage as urine is in contact with the tumor and can be collected in an easy, non-invasive and repeatable way. Urinary exfoliated cancer cells and secreted nucleic acids mirror bladder tumors along each step of cancer development [21]. In particular, the study of relevant mutations in urine has the potential to become a reliable tool for diagnostic/prognostic purposes [22–24].

The aim of our study was to search simultaneously for mutations in *TERT*, *FGFR3*, *PIK3CA*, and *RAS* genes in the urinary exfoliated cells of a consecutive series of BC patients, both NMIBC and MIBC, and to evaluate their role as predictors of recurrence, progression, and survival. In a subgroup of 81 NMIBC patients, who have provided additional urine samples during their follow-up (for a total of 324 samples), mutations were repeatedly screened. To the best of our knowledge, this represents the largest number of mutations simultaneously investigated in urine, both at diagnosis and during clinical surveillance, for their association with BC.

## RESULTS

### Patient characteristics

Baseline patients’ characteristics are reported in Table 1. The cohort included 230 (90.2%) NMIBC patients most of whom were classified as high-risk (40%) and received adjuvant intravesical therapy (52.2%). Recurrence and progression were experienced by 38.3% and 4.3% of patients, respectively.

**Table 1:** Baseline characteristics of the patients with NMIBC or MIBC and information at follow-up

VARIABLES	NMIBC (n= 230)	MIBC (n= 25)
	N (%)	N (%)
**Age** (years)		
Mean ± SD	63.8 ± 8.0	64.7 ± 8.6
Range	40.0-74.9	40.2-73.5
**Smoking Status**		
Never	22 (9.6)	3 (12.0)
Former	121 (52.6)	9 (36.0)
Current	87 (37.8)	13 (52.0)
**T stage**		
Tx	2 (0.9)	-
Ta	147 (63.9)	-
Tis	7 (3.0)	-
T1	74 (32.2)	-
T2	-	23 (92.0)
T3+T4	-	2 (8.0)
**Grading (1973)**		
G1	70 (30.4)	-
G2	99 (43.0)	1 (4.0)
G3	61 (26.6)	24 (96.0)
**Grading (2004)**		
Low grade	125 (54.3)	-
High grade	105 (45.7)	25 (100.0)
**Tumor size (cm)**		
<3	168 (73.0)	7 (28.0)
≥3	62 (27)	18 (72.0)
**Risk**		
Low-risk	60 (26.1)	-
Intermediate Risk	78 (33.9)	-
High-risk	92 (40.0)	-
**Recurrence**		
No	142 (61.7)	-
Yes	88 (38.3)	-
**Number of recurrences**		
1	56 (63.6)	-
2	21 (23.9)	-
≥3	11 (12.5)	-
**Progression to MIBC**		
No	220 (95.7)	-
Yes	10 (4.3)	-
**Therapy**		
No	110 (47.8)	22 (88.0)
Yes	120 (52.2)	3 (12.0)
**Type**		
BCG	67 (55.8)	-
Chemotherapy	53 (44.2)	3 (100.0)
**Cystectomy**		
No	209 (90.9)	6 (24.0)
Yes	21 (9.1)	19 (76.0)
**Disease relapse after cystectomy**		
No	14 (66.7)	9 (47.4)
Yes	7 (33.3)	10 (52.6)
**Type**		
Local	3 (42.8)	3 (30.0)
Distal	2 (28.6)	1 (10.0)
Local+Distal	2 (28.6)	6 (60.0)
**Status at follow-up**		
Alive	198 (86.1)	9 (36.0)
Dead of all causes	32 (13.9)	16 (64.0)
Dead of bladder cancer	11 (4.8)	9 (36.0)

Twenty-five (9.8%) primary MIBC patients were also enrolled, 19 of which underwent radical cystectomy.

At a median follow-up of 5.83 years (range 1.21-8.54 years), cancer-specific and overall mortality rates in primary NMIBC patients were 4.8% (n=11) and 13.9% (n=32), respectively, and in primary MIBC cases were 36% (n=9) and 64% (n=16), respectively.

### Mutation analysis in urinary exfoliated cells at diagnosis

Among the 230 urines obtained from the primary NMIBC cases, 159 samples had at least one mutation, thus sensitivity of tumor detection was 69%. *TERT* mutations were found in 119 (52%) urine samples while 95 (41.6%) had a *FGFR3* mutation. Sensitivity of the combination of both genes was 67%. Hence mutations in the *RAS* and *PIK3CA* genes only added 2% to the sensitivity (Table 2). The most common *TERT* mutations were the -124C>T (n=117) and -146C>T (n=19), with two subjects presenting both mutations simultaneously, while only five patients had mutations at -124C>A and -138_139CC>TT. The most common *FGFR3* mutation was S249C (46%), followed by Y375C, K652M, K652E, and R248C (45% together). In 12 subjects, two simultaneous *FGFR3* mutations were detected while two subjects presented three mutations. Occurrence of more than one mutation for *FGFR3* and *TERT* has been found before [22, 25] and is probably due to either tumor heterogeneity or the presence of multiple tumor clones in the bladder. Mutations in *PIK3CA* and *RAS* genes were detected in 13% and 4.8% of urine samples, respectively. For *PIK3CA*, the most frequent mutation was E545K (44%). Finally, for *RAS* genes, the majority of mutations were in *KRAS* G12V (25%). Distribution of mutations for each investigated gene is reported in Supplementary Figure S1.

**Table 2:** Distributions of mutations of investigated genes in urinary exfoliated cells according to clinical and demographic characteristics of NMIBC patients

Variables	*TERT*		*FGFR3*		*PIK3CA*		*Ras*	
	No Mutations N (%)	≥1 Mutations N (%)	No Mutations N (%)	≥1 Mutations N (%)	No Mutations N (%)	≥1 Mutations N (%)	No Mutations N (%)	≥1 Mutations N (%)
**All patients**	110 (48%)	119 (52%)	133 (58.4)	95 (41.6)	200 (87.0)	30 (13.0)	219 (95.2)	11 (4.8)
**Age** (years) Mean ± SD	63.1 ± 8.1	64.7± 7.9	63.7±8.3	63.9±7.6	64.1±7.9	61.5±8.4	63.7±8.0	64.4±8.0
	p= 0.13		p= 0.85		p= 0.10		p= 0.78	
**Smoking status**								
Never	42 (38.2%)	44 (37.0%)	50 (37.6%)	34 (35.8%)	76 (38.0%)	10 (33.3%)	81 (37.0%)	5 (45.5%)
Former	59 (53.6%)	62 (52.1%)	70 (52.6)	52 (54.7%)	104 (52.0%)	18 (60.0%)	117 (53.4%)	5 (45.5%)
Current	9 (8.2%)	13 (10.9%)	13 (9.8%)	9 (9.5%)	20 (10.0%)	2 (6.7)	21 (9.6%)	1 (9.0%)
	p= 0.78		p= 0.95		p= 0.68		p= 0.84	
**Grading (1973)**								
G1	48 (43.7%)	22 (18.5%)	35 (26.3%)	34 (35.8%)	57 (28.5%)	13 (43.3%)	66 (30.1%)	4 (36.4%)
G2	47 (42.7%)	51 (42.9%)	50 (37.6%)	48 (50.5%)	86 (43.0%)	13 (43.3%)	94 (42.9%)	5 (45.4%)
G3	15 (13.6%)	46 (38.6%)	48 (36.1%)	13 (13.7%)	57 (28.5%)	4 (13.4%)	59 (27.0%)	2 (18.2%)
	**p<0.0001**		**p= 0.01**		p= 0.13		p= 0.80	
**Grading (2004)**								
Low grade	75 (68.2%)	50 (42.0%)	63 (47.4%)	60 (63.2%)	103 (51.5%)	22 (73.3%)	117 (53.4%)	8 (72.7%)
High grade	35 (31.8%)	69 (58.0%)	70 (52.6%)	35 (36.8%)	97 (48.5%)	8 (26.7%)	102 (46.6%)	3 (27.3%)
	**p <0.0001**		**p= 0.02**		**p= 0.03**		p= 0.21	
**Tumour size**								
<3cm	86 (78.2%)	81 (68.1%)	100 (75.2%)	66 (69.5%)	147 (73.5%)	21 (70.0%)	161 (73.5%)	7 (63.6%)
≥3cm	24 (21.8%)	38 (31.9%)	33 (24.8%)	29 (30.5%)	53 (26.5%)	9 (30.0%)	58 (26.5%)	4 (36.4%)
	p= 0.09		p= 0.34		p= 0.69		p= 0.47	
**Stage**								
Ta	82 (74.6%)	64 (53.8%)	78 (58.6%)	67 (70.5%)	126 (63.0%)	21 (70.0%)	139 (63.5%)	8 (72.7%)
T1	24 (21.8%)	50 (42.0%)	48 (36.1%)	26 (27.4%)	65 (32.5%)	9 (30.0%)	71 (32.4%)	3 (27.3%)
Tis	2 (1.8%)	5 (4.2%)	5 (3.8%)	2 (2.1%)	7 (3.5%)	0	7 (3.2%)	0
Tx	2 (1.8%)	0	2 (1.5%)	0	2 (1.0%)	0	2 (0.9%)	0
	**p= 0.01**		p= 0.22		p= 0.66		p= 0.88	
**Risk**								
Low Risk	40 (36.4%)	20 (16.8%)	31 (23.3%)	28 (29.5%)	49 (24.5%)	11 (36.7%)	57 (26.0%)	3 (27.3%)
Intermediate Risk	40 (36.4%)	37 (31.1%)	41 (30.8%)	36 (37.9%)	68 (34.0%)	10 (33.3%)	73 (33.4%)	5 (45.4%)
High Risk	30 (27.2%)	62 (52.1)	61 (45.9%)	31 (32.6%)	83 (41.5%)	9 (30.0%)	89 (40.6%)	3 (27.3%)
	**p <0.0001**		p= 0.13		p= 0.31		p= 0.62	
**Recurrence**								
No	73 (66.4%)	69 (58.0%)	90 (67.7%)	52 (54.7%)	126 (63.0%)	16 (53.3%)	132 (60.3%)	10 (90.9%)
Yes	37 (33.6%)	50 (42.0%)	43 (32.3%)	43 (45.3%)	74 (37.0%)	14 (46.7%)	87 (39.7%)	1 (9.1%)
	p= 0.19		**p= 0.05**		p= 0.30		**p= 0.04**	
**Number of recurrences**								
1	27 (73.0%)	28 (56.0%)	32 (74.4%)	23 (53.5%)	47 (63.5%)	9 (64.3%)	55 (63.2%)	1 (100.0%)
≥2	10 (27.0%)	22 (44.0%)	11 (25.6%)	20 (46.5%)	27 (36.5%)	5 (35.7%)	32 (36.8%)	0
	p= 0.10		**p= 0.04**		p= 0.96		p= 0.45	
**Progression to MIBC**								
No	109 (99.1%)	110 (92.4%)	125 (94.0%)	93 (97.9%)	191 (95.5%)	29 (96.7%)	209 (95.5%)	11 (100.0%)
Yes	1 (0.9%)	9 (7.6%)	8 (6.0%)	2 (2.1%)	9 (4.5%)	1 (3.3%)	10 (4.5%)	0
	**p= 0.01**		p= 0.16		p= 0.77		p= 0.47	
**Survival at follow-up**								
Alive (or died due to causes other than BC)	109 (99.1%)	109 (91.6%)	124 (93.2%)	93 (97.9%)	190 (95.0%)	29 (96.7%)	209 (95.5%)	10 (90.9%)
Dead	1 (0.9%)	10 (8.4%)	9 (6.8%)	2 (2.1%)	10 (5.0%)	1 (3.3%)	10 (4.5%)	1 (9.1%)
	**p= 0.01**		p= 0.11		p= 0.70		p= 0.49	

Significant results in bold.

**Figure 1:**
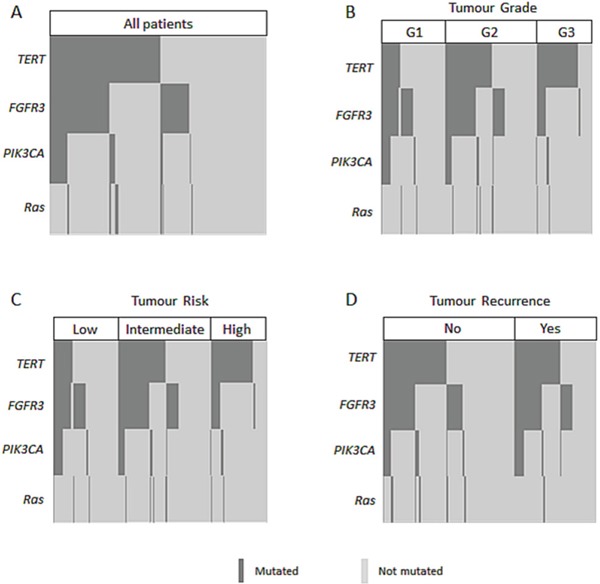
Concurrent and mutually exclusive mutations in NMIBC patients at diagnosis **A.** and stratified according to grade **B.**, risk **C.**, or recurrence **D.**

The relationship between mutations observed in urine and clinical factors is reported for NMIBC in Table 2. *TERT* mutations significantly correlated with the presence of high-risk, high-grade tumors (for both p<0.0001) and progression to muscle-invasive disease (p=0.01). *FGFR3* was associated with low-grade tumors (p=0.02) and event of recurrence/number of recurrences (p=0.05, p=0.04, respectively). *PIK3CA* mutations were more common in urine collected from low-grade tumors (p=0.03). Despite the low frequency of *RAS* mutations, an association was also seen between mutated *RAS* and no recurrence (p=0.04). The distribution of mutations at diagnosis with respect to the different genes and relation to tumor grading, risk, and recurrence are highlighted in Figure 1. As shown, *TERT*, *FGFR3*, and *PIK3CA* mutations overlap to some extent.

In Supplementary Table S1 the sensitivities of the assays for detection of muscle-invasive primary tumors is shown. In this group, 80% of the urine samples presented with a *TERT* mutation while other genes were less frequently mutated (<20%). We further investigated the possible association of the mutations and clinical parameters for MIBC patients. However, although there were some statistically significant relations, the numbers were too low to draw conclusions.

### Overall and cancer survival

**Figure 2:**
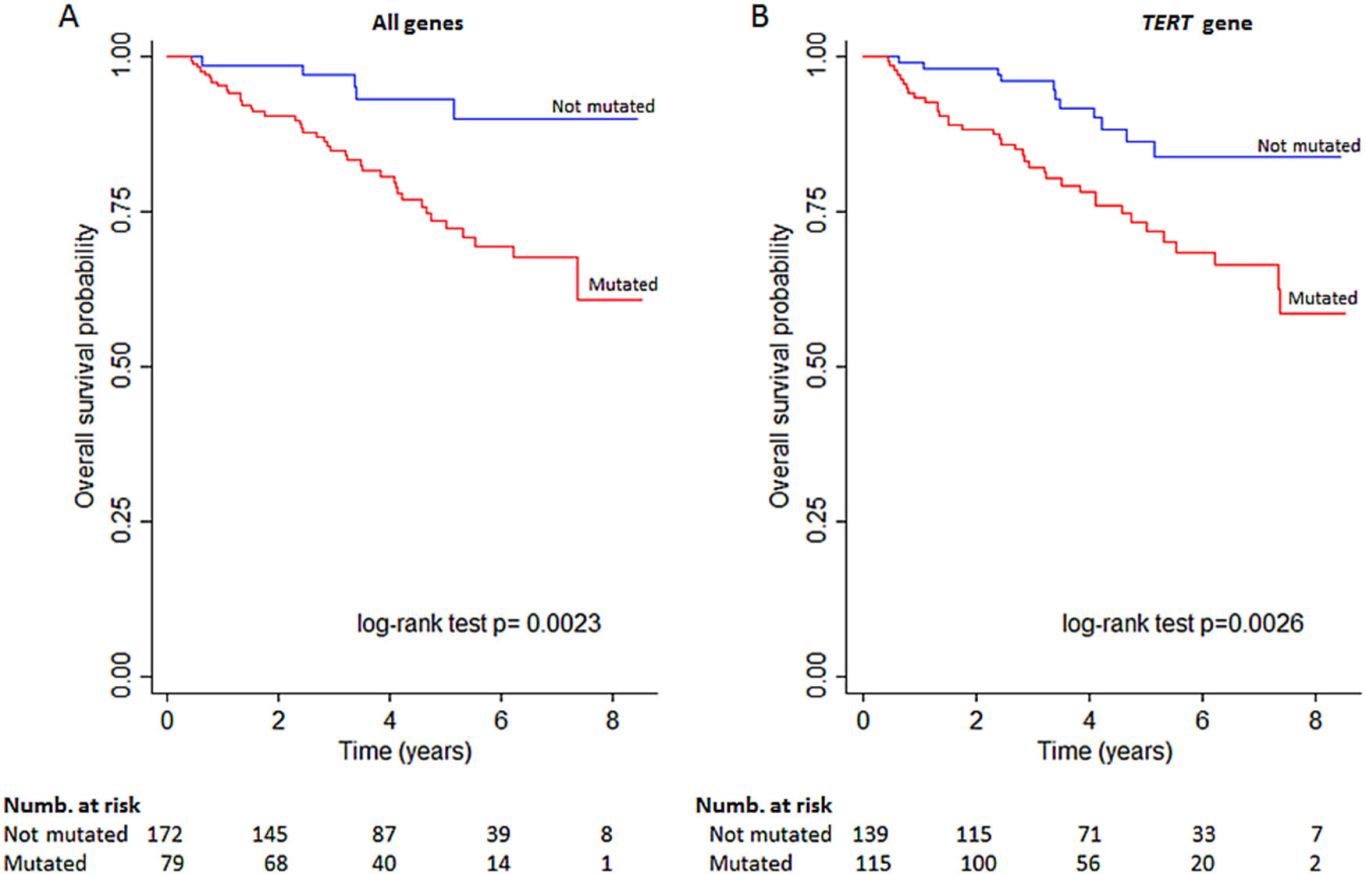
Kaplan-Meier curve showing OS for A. subjects stratified for combined genes mutational status or B. TERT mutations alone.

A higher percentage of patients who died either from any cause or cancer carried mutations in the analyzed genes at diagnosis (89.4% and 88.5%, respectively, data not shown). As presented in Supplementary Table S2, there was a significant association with OS in the adjusted model for *FGFR3* mutations (p=0.04). Associations with OS were also noted with different combinations of genetic mutations. Figure 2 depicts Kaplan-Meier curves for all mutations combined (log-rank p=0.002) and *TERT* mutations only (log-rank p=0.003). No significant associations were observed between the mutations and cancer-specific survival.

### Association of the presence of mutations at diagnosis and the occurrence of recurrences

In the NMIBC group, 88 subjects out of 230 (38%) experienced a recurrence during the clinical follow-up; among them, 56 recurred only once while the others manifested two or more recurrences. Of all patients who recurred, 28 (32%) had one gene mutated at diagnosis while 34 (40%) presented two or more mutations. Thirty-one subjects had more than one recurrence (with a maximum of five) with 27 of them (87%) presenting one or more mutations. Among the remaining 142 non-recurring patients, 23 (16%) had at least one mutation in a gene while 21 (15%) had more genes mutated simultaneously.

There was a consistently significant increase in the number of mutations in concomitance with an increased number of recurrences when genes were analyzed in combination or individually (OR: 2.05 95% CI: 1.05-3.98, p=0.03). Although combining mutations in all genes significantly associated with recurrences, stronger associations were observed especially when mutations in the *FGFR3* and *TERT* genes were combined (OR: 2.51 95% CI: 1.26-5.00, p=0.01, Supplementary Table S3).

### Detection of recurrences in NMIBC patients under surveillance

For 81 NMIBC patients, four additional urine samples were periodically collected over approximately three years after recruitment. There were no major differences in the investigated clinical parameters between this subgroup and the whole cohort. Within this subgroup, 34 subjects underwent intravesical BCG treatment, 19 underwent intravesical chemotherapy, while the remaining 28 did not receive further treatment beyond TURB. Five of these patients died of causes other than BC during follow-up, all after recurrence and one after progression, and all of them presented mutations in their analyzed urine samples, either at diagnosis or follow-up.

In the first follow-up mutation analysis after diagnosis, the frequency of mutations dropped from 68% of subjects to 19%, still being mutations *TERT* mutation -124C>T the most frequent. The number of *TERT* mutations remained similar in the exfoliated cells from the collections that followed (data not shown).

Thirty-five subjects (43.2%) had a recurrence; of them, 27 (77%) presented one or more gene mutations. All 14 patients with multiple recurrences, except one, presented at least one mutation. Multivariate analyses (Table 3) confirmed the association between multiple gene mutations and the number of recurrences. Stronger associations were observed for *TERT* and *FGFR3* mutations combined with number of recurrences (OR: 4.51, 95% CI: 1.27-16.06, p= 0.02 at diagnosis; OR: 4.54 95% CI: 1.23-16.79, p= 0.02 at diagnosis, and follow-up). When *FGFR3* and *TERT* gene mutations were observed in the diagnostic and a follow-up urine sample, the sum of their mutations detected at diagnosis and follow-up were more strongly associated either with the event of recurrence or with the number of recurrences than at diagnosis alone. Of those 29 patients with a mutation at diagnosis who did not recur, 23 (79.3%) did not present any further mutations over the four follow-up samplings. The distribution of mutations in *FGFR3* and *TERT* in respect to recurrences in repeated samples is depicted by some examples in Supplementary Figure S2.

**Table 3:** Mutations in repeated urine samples collected during follow-up in the subgroup of NMIBC patients (n= 81) in association with event(s) of recurrence

Genes	At diagnosis				At diagnosis +follow-up			
	Recurrence yes/no		Number of recurrences		Recurrence yes/no		Number of recurrences	
	OR (95% CI)	p-value	OR (95% CI)	p-value	OR (95% CI)	p-value	OR (95% CI)	p-value
**All genes combined**								
1 gene mutated	1.71 (0.48-6.09)	0.41	1.91 (0.56-6.52)	0.30	0.99 (0.19-5.14)	0.99	1.02 (0.21-4.83)	0.98
2 or more	3.19 (0.90-11.28)	0.07	4.11 (1.23-13.69)	**0.02**	3.16 (0.81-12.23)	0.10	3.80 (1.06-13.58)	**0.04**
***TERT+FGFR3+PIK3CA***								
1 gene mutated	1.78 (0.52-6.15)	0.36	1.98 (0.60-1.26)	0.26	2.02 (0.44-9.29)	0.37	1.87 (0.45-7.72)	0.39
2 or more	3.30 (0.90-12.11)	0.07	4.31 (1.26-14.76)	**0.02**	3.40 (0.85-13.63)	0.08	4.07 (1.11-14.84)	**0.03**
***TERT+FGFR3+Ras***								
1 gene mutated	1.86 (0.55-6.35)	0.32	2.07 (0.63-6.73)	**0.23**	0.86 (0.17-4.40)	0.86	0.92 (0.20-4.31)	0.91
2 or more	3.17 (0.864-11.63)	0.08	4.21 (1.23-14.35)	**0.02**	3.51 (0.89-13.77)	0.07	4.13 (1.15-14.84)	**0.03**
***TERT+FGFR3***								
1 gene mutated	1.91 (0.57-6.37)	0.29	2.11 (0.66-6.73)	0.21	1.75 (0.39-7.89)	0.47	1.67 (0.41-6.86)	0.48
2 or more	3.32 (0.86-12.86)	0.08	4.51 (1.27-16.06)	**0.02**	3.87 (0.94-15.87)	0.06	4.54 (1.23-16.79)	**0.02**
***TERT***	2.42 (0.79-7.39)	0.12	3.01 (1.06-8.59)	**0.04**	1.91 (1.13-3.22)	**0.01**	1.65 (1.14-2.39)	**0.01**
***FGFR3***	2.01 (0.72-5.61)	0.18	2.27(0.86-6.02)	0.10	1.89 (1.04-3.46)	**0.04**	1.54 (1.05-2.26)	**0.02**
***PIK3CA***	1.30 (0.33-5.14)	0.71	1.35 (0.39-4.76)	0.64	1.36 (0.68-2.77)	0.38	1.28 (0.75-2.21)	0.37
***Ras***	1.59 (0.08-29.76)	0.76	1.28(0.099-22.05)	0.78	1.56 (0.52-4.70)	0.43	1.38 (0.52-3.67)	0.52

Adjusted for age, smoking status, grade, stage and therapy. Significant results in bold.

**Figure 3:**
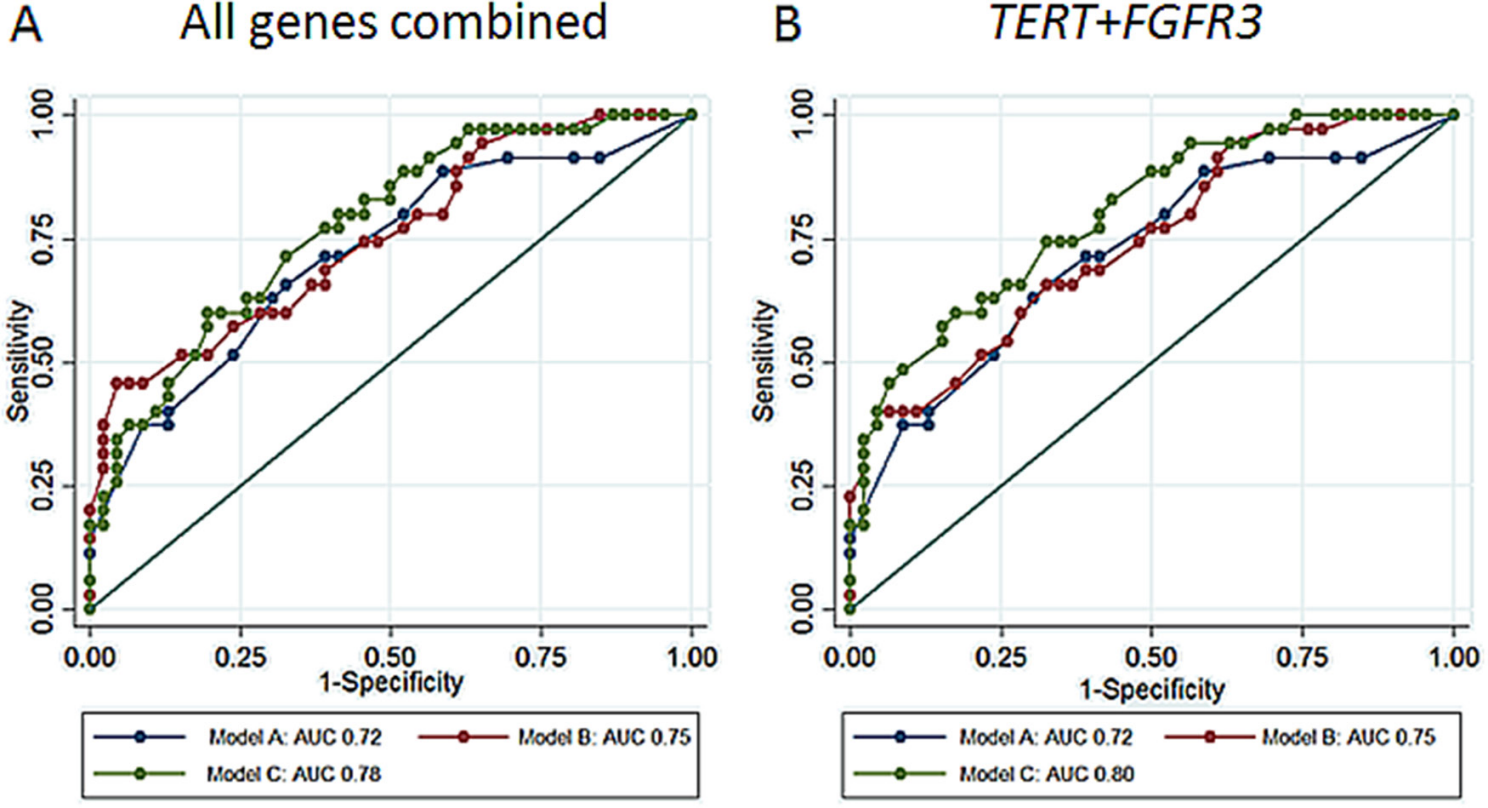
**A.** AUC for a set of BC risk factors (age, smoking status, and risk of recurrence, model A) and the same set of factors and mutational status of all genes at diagnosis (model B), and at diagnosis plus follow-up (model C). **B.** AUC for the same models but considering the mutational status of TERT and FGFR3 genes only.

A prediction model for the presence of a recurrence was developed including age, smoking status, and risk of recurrence as covariates (Model A). Then, the mutational status as a predictor (Model B) and, finally, the mutational status during follow-up (Model C) were added each time to Model A. Considering all genes together, the area under curve (AUC) of the model increased from 0.72 (95% CI: 0.61-0.83, Model A) to 0.75 (95% CI: 0.65-0.86, Model B) and to 0.78 (95% CI: 0.68-0.88, Model C). Using only the genes *FGFR3* and *TERT*, model C reached an AUC of 0.80 (95% CI: 0.71-0.89, model C). Receiver operating characteristic (ROC) curves for the different models are reported in Figure 3A and 3B, respectively, for all genes together or the *FGFR3* and *TERT* combination. Adding the information of mutational status at diagnosis and follow-up, the predictive accuracy of the model was 8% higher when compared to that of the models usually used in clinical practice.

## DISCUSSION

In the present study, we used urine analysis to diagnose primary and recurrent bladder tumors. We also looked at associations between mutations in urine at first diagnosis and clinical parameters. We selected six genes (*FGFR3*, *PIK3CA*, *RAS* family genes, and *TERT* promoter) and determined their mutation profile in urine samples of BC patients, first at diagnosis, prior to treatment, and secondly, for NMIBC patients, during their follow-up. The tests detected 70% of the first presentation NMIBC. Sensitivity for *TERT* and *FGFR3* were 52% and 42%, respectively. The distribution of mutations among genes is similar to other previous studies on tumor [22, 26–29] and voided urine [22, 30, 31], although *TERT* mutations are lower [22, 32]. Two very recent studies have reported the same *TERT* mutation frequencies in urine of BC patients as us by different methodologies [26, 33]. In the small group of MIBC patients, sensitivity of the *TERT* assay was 80% while mutations in the other genes were less represented, in line with the distribution found in other studies [22]. Both *TERT* and *FGFR3* mutations were related to clinical parameters: *TERT* mutations with high-risk, high-grade NMIBC, as well as with progression to MIBC and *FGFR3* with low-grade tumors. Mutations in the *FGFR3* gene have been previously found in 70% of the low-grade BC and are usually associated with a favorable prognosis [25, 34]. Patients with low-grade tumors are those at lower risk of progression, and the presence of an *FGFR3* mutation has been associated with such behavior earlier [34, 35]. For *TERT*, mutations in urine were not previously observed in association with clinical parameters.

The same set of mutations was analyzed in exfoliated cells of samples collected from NMIBC patients during clinical surveillance. To our knowledge, this is the largest series of mutation analyses in multiple genes in repeated observations over time (for an overall number of 405 analyses). As expected, after the removal of the primary tumors with TURB and subsequent intravesical therapy, the number of patients with mutations in urinary cells was considerably lower. Considering the potential of *FGFR3* and *TERT* mutations at diagnosis and during follow-up for the prediction of recurrences, we explored if their implementation in clinical practice could improve the risk models currently used by urologists [4]. According to our results, the analysis of mutational status could improve the recurrence risk as measured by the EORTC risk tables with 8%. Similar findings were observed for *FGFR3* only in repeated urine samples collected over three years by Zuiverloon and colleagues [30]. We detected an association between the presence of multiple mutations and the number of recurrences, in particular, when *TERT* and *FGFR3* mutations were combined. Even though *TERT* promoter mutations were more frequent than *FGFR3* ones, the alterations at the two loci occurred together more frequently than per chance.

Considering the importance of detecting recurrences in patients with a low grade NMIBC, we have repeated analyses only in those patients belonging to this category. Interestingly, the frequency of mutations detected (combining all genes or considering them individually) and the associations previously described remained fairly similar to the whole set of NMIBC patients at diagnosis, or in the subset of those who provided multiple urine samples over time. In particular, in this last category, subjects with multiple recurrences were all mutated. This is particularly important considering that low grade tumors will experience more recurrences (as we also observed in our study population, i.e. 46% vs. 38%) and in routine investigations, like cytology, have a lower sensitivity in comparison to high-grade ones.

We are aware that the present study is affected by some limitations. First, there are inherent problems with urinary testing. As volume and stage/grade of BC can vary vastly, DNA testing must be able to deal with a small amount of exfoliated cells in urine. This is particularly true in post-diagnostic repeated sample collections, where the primary tumor has been removed and treatments may have occurred. In this respect, preliminary experiments were performed to investigate the sensitivity of the mutation detection assay by single nucleotide primer extension (SNaPshot) sequencing. Serial dilutions of samples at increasing concentrations of DNA from a healthy subject and a human cell line derived from a transitional cell carcinoma of the renal pelvis (UM-UC-14) were tested to identify the optimal concentration. The SNaPshot assay has proven to be an extremely sensitive technique able to detect even 1% of mutant alleles in a given sample, either with pre-amplification or without (data not shown). Moreover, in this study, no pre-amplification of the DNA of the samples was necessary. Urine represents a very dynamic fluid, and its composition can be affected by fluid status, renal disease, infection, and urinary tract instrumentation posing a disadvantage for the identification of a good marker. However, gene-based urinary biomarkers are more sensitive and specific as they detect cancer-related changes which are less likely to be affected by inflammatory and other benign conditions compared with protein-based detection [36].

From the point of view of the study design, repeated samplings of the subgroup of patients were performed not always in concomitance with the detection of a recurrence. Thus, we could not detect the presence of a mutation before or simultaneously with an event of recurrence and establish a clear relationship. Finally, our study is also limited since it is based on males only. BC is almost three times more common in men than in women; therefore, it is relatively simple to collect male patients. Although most likely some differences exist between genders for mutation detection and clinical outcomes [37, 38], we were unable to explore this. However, performing the assay only on men greatly reduced the variability.

Nevertheless, several strengths of this study must also be acknowledged: amongst them, the prospective design, the large number of samples analyzed, and the possibility of analyzing the mutational status of patients during follow-up. The heterogeneity of the population enrolled might be seen as a drawback, but in our opinion it reflects the complexity of BC, strengthening our findings. In addition, the analyses of all mutations were performed simultaneously by multiplexing the reactions. This is a very useful approach to screen a vast number of mutations at once, allowing us to save the biological material and always using the same experimental conditions at relatively low costs [12]. Moreover, urine is in direct contact with BC and sediment cells collected from voided samples are an ideal source of DNA because they are readily available, and the sampling is not invasive [39].

Most patients diagnosed with BC have NMIBC (75%) that are amenable to TURB and have a good five-year survival rate (80-90%). However, 70% of subjects will have at least one recurrence within five years, and some will even recur after 15 years of surveillance. Although most NMIBC patients have recurrences of low-stage and grade, progression to MIBC is observed in 10-20%, leading to an entirely different scenario characterized by a worse prognosis despite radical treatments. The high recurrence rate and risk of progression to MI disease necessitates the frequent monitoring of NMIBC patients by performing periodic endoscopic procedures, with huge costs for the health system and discomfort for patients themselves [40–42]. To address this issue, in the era of the so-called “liquid biopsies”, research is focusing on the identification of molecular markers associated with the presence of BC [43, 44]. The ultimate goal would be to develop a urine-based biomarker to reduce the cystoscopy frequency and improve the patients' quality of life without jeopardizing their safety. Such tests should be easy-to-perform, reproducible, and simple to interpret. In this respect, from our study the panel of investigated gene mutations in voided urine samples may have the potential to be helpful in the diagnosis and surveillance of BC patients. In particular, mutations in the *TERT* promoter and *FGFR3* are the most frequently detected, with rates comparable to those of other studies and reflecting the situation in primary tumor tissue. Larger prospective studies from international consortia will provide the adequate study population size, including specific subgroups of patients with specific features of this cancer, to investigate the promising markers emerging. It can be expected that combinations of different molecular and histopathological biomarkers will be introduced into the clinical setting in the near future [45].

## MATERIALS AND METHODS

### Study population

The study population included 255 newly diagnosed, histologically confirmed cases of BC recruited at Città della Salute e della Scienza Hospital, Turin (Italy), between 2006 and 2012. All subjects were men aged 40–75 years, living in the Turin metropolitan area that signed an informed consent form. Clinical and socio-demographic information were collected from all patients. Staging and grading were performed by expert uropathologists according to the tumor-node-metastases (TNM) 2002 classification and both the 1973 and 2004 World Health Organization (WHO) classifications. Patients were classified according to current European Organization for Research and Treatment of Cancer (EORTC) risk tables [46]. Recurrence was defined as the occurrence of any CIS and/or papillary Ta-T1 tumor during follow-up, whereas the finding of T>1 was considered as progression. Progression for MIBC patients was defined as the presence of a local, distal or local-distal extravesical disease. Causes of death were retrieved from local demographic offices and death certificates. All treatments were recorded. At 3-months follow-up, urine cytology and cystoscopy with TUR of all visible lesions and a biopsy mapping when appropriate were undertaken. Successively, follow-up was scheduled as per European Association of Urology (EAU) guidelines [5]. Subjects were defined as former smokers if they had quitted smoking ≥1 year before the enrollment. This study was performed according to the principles of the Declaration of Helsinki and in agreement with ethical requirements. An internal ethical review board at Human Genetics Foundation of Turin (HuGeF committee/17-11-2011) approved the study.

### Urine sample collection and DNA isolation from exfoliated cells

All subjects recruited in the study provided a urine sample at the time of diagnosis before any treatment. A subgroup of patients (n=81) under surveillance after diagnosis provided additional urine samples, approximately every six months from the recruitment, for a total of four collections. Freshly voided urine (10-100 mL) was collected before cystoscopy and stored at 4°C until their processing. Cells were pelleted by centrifugation for 10 minutes at 1,500 rpm and washed twice with 10 mL phosphate buffered saline (PBS). Finally, the cell pellet was stored at −80°C until DNA isolation. DNA was extracted using the QIAamp DNA Mini Kit (Qiagen) according to the manufacturer's protocol. DNA concentration was measured with Quant-iT High-Sensitivity DNA Assay Kit (Life Technologies), and all samples were diluted to a fixed concentration of 5 ng/μl.

### Mutation analyses

The mutation assays were performed as described by van Oers and colleagues [47]. Briefly, we performed three multiplex polymerase chain reactions (PCR) of the exons containing the most common mutations in the genes of interest. Each multiplex PCR reaction was performed in a total volume of 10 μl containing KAPA2G Robust HotStart ReadyMix 2X (KAPA Biosystems), 0.5 μl of the appropriate primer combination, and 5 ng of genomic DNA as template. Thermal cycling conditions consisted of initial denaturation at 95°C for 3 minutes, followed by 40 cycles each consisting of 95°C for 15 seconds, 55°C for 15 seconds, and 72°C for 20 seconds. The final elongation step was 72°C for 10 minutes. Unincorporated primers and deoxynucleotide triphosphates were removed from PCR products by addition of 2 units of Exonuclease I (*ExoI*) and 1.5 units of Shrimp Alkaline Phosphatase (SAP, USB Corporation) at 37°C for 60 minutes and 72°C for 15 minutes.

PCR products were subsequently analyzed for mutations using probes for each of the possible mutation sites and SNaPshot Multiplex Kit (Life Technologies). The mutation detection reactions were performed in a total volume of 10 μl containing 2.5 μl of SNaPshot Multiplex Ready Reaction Mix, 2 μl of BigDye Sequencing buffer, 1 μl of probe mix, and 1 μl of SAP/ExoI treated PCR product. Extension reactions consisting of 35 cycles of denaturation at 96°C for 10 seconds and annealing/extension at 58.5°C for 40 seconds were performed in a thermal cycler. After extension, the excess of labelled dideoxynucleotide triphosphates was removed by treatment with 1 unit of SAP at 37°C for 60 minutes and 72°C for 15 minutes. Extended primers were denatured at 95°C for 4 minutes and separated by capillary electrophoresis on an automatic sequencer ABI PRISM 3130 XL Genetic Analyzer (Life Technologies). The presence or absence of a mutation was indicated by the fluorescent label on the incorporated nucleotide. Data were analyzed using GeneScan Analysis Software version 3.7 (Life Technologies) and GeneMarker Software version 2.6 (SoftGenetics LLC).

The first multiplex assay identifies simultaneously seven *PIK3CA* hotspot mutations (E542K, E545A, E545G, E545K, E545Q, H1047R and H1047L) and four *TERT* mutations (−124C>T (G>A), -124C>A (G>T), -138_139CC>TT (GG>AA) and -146C>T (G>A)). A second multiplex PCR detects the most frequent *FGFR3* mutations in three regions that comprise the following codon mutations: R248C and S249C (exon 7); G372C, S373C, Y375C, G382R, A393E (exon 10); and K652M, K652T, K652E, K652Q (exon 15). Somatic mutations in the *HRAS*, *KRAS* and *NRAS* genes in BC affect codons 12, 13 and 61. To facilitate detection of *RAS* mutations, we used a multiplex *RAS* mutation assay that screens for 19 mutations simultaneously, representing 96% of all possibly known mutations in the three *RAS* genes [12].

### Statistical analysis

Descriptive statistics of the baseline characteristics of the patients were reported using means and percentages, for NMIBC and MIBC separately.

The relationships between mutational status and clinical-demographic characteristics of patients were analyzed by T-test, Chi-square test, and Fisher exact test.

Follow-up time was considered time from diagnosis to death or loss of follow-up, depending on which came first. In the OS analysis all deaths were considered as events; in the cancer survival analysis, the events were only deaths of cancer. Kaplan-Meier plots were used to estimate the probability of overall and cancer survival according to the mutational status at diagnosis (log-rank test was performed to compare the curves).

Univariate logistic regression models were used considering the different clinical and demographic factors as predictors. The models were estimated for each outcome of interest, to identify the confounders to be included in the subsequent multivariate analysis.

A logistic regression model was fitted to estimate the odds ratio (OR) of recurrence comparing the several categories of mutational status for each gene and the genes combined. An ordered logistic regression model was also applied to estimate the impact of mutational status on the number of recurrences (0, 1, ≥2). In both analyses two different models were used: one unadjusted, and another adjusted for age, smoking status, stage, grade and therapy.

Finally, different prediction models for recurrence were built: 1) a model including age, smoking status, and risk of recurrence as predictors (Model A); 2) Model A with the additional predictor “mutational status at diagnosis” (Model B); and 3) Model A with the additional predictor “mutational status at diagnosis and follow-up” (Model C). ROC curves were plotted and AUC were calculated for each model to evaluate their performance prediction (range between 0.5 -random discrimination- and 1.0 -perfect discrimination-).

All analyses were performed using STATA version 13 (StataCorp, LP).

## SUPPLEMENTARY MATERIAL FIGURES AND TABLES




