# Supplementary material for: Detection of multiple mutations in urinary exfoliated cells from male bladder cancer patients at diagnosis and during follow-up
Source: Oncotarget. 2016 Sep 7;7(41):67435–48. doi: 10.18632/oncotarget.11883 (PMC5341887; doi:10.18632/oncotarget.11883)
Supplement: Supplementary file 1 [file oncotarget-07-67435-s001.pdf]

Detection of multiple mutations in urinary exfoliated cells from male bladder cancer patients at diagnosis and during follow-up

SUPPLEMENTARY FIGURES AND TABLES

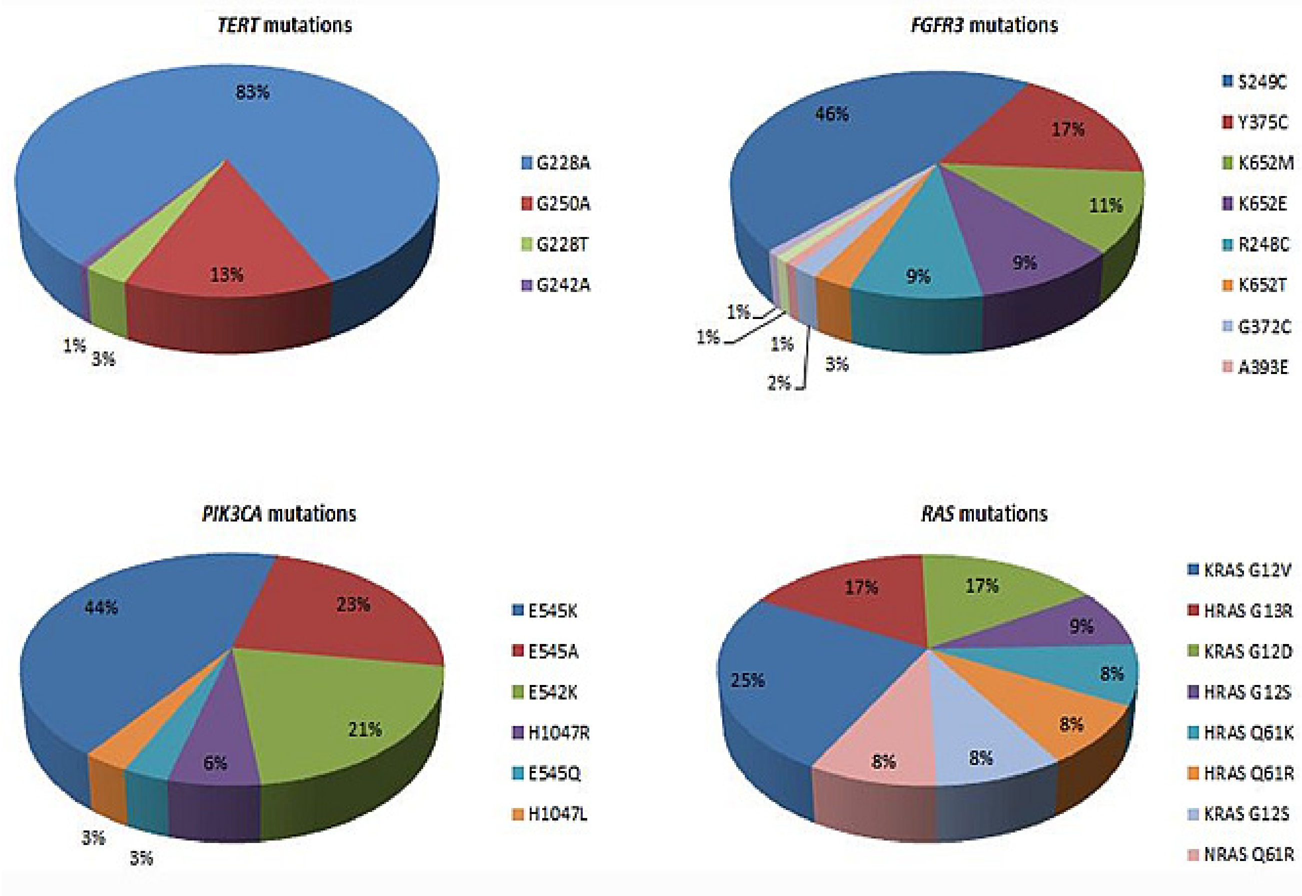

Supplementary Figure S1: Pie charts showing the distribution of mutations in individual genes in the study population of BC patients. Out of 41 detectable mutations, 22 were found in urine samples analysed.

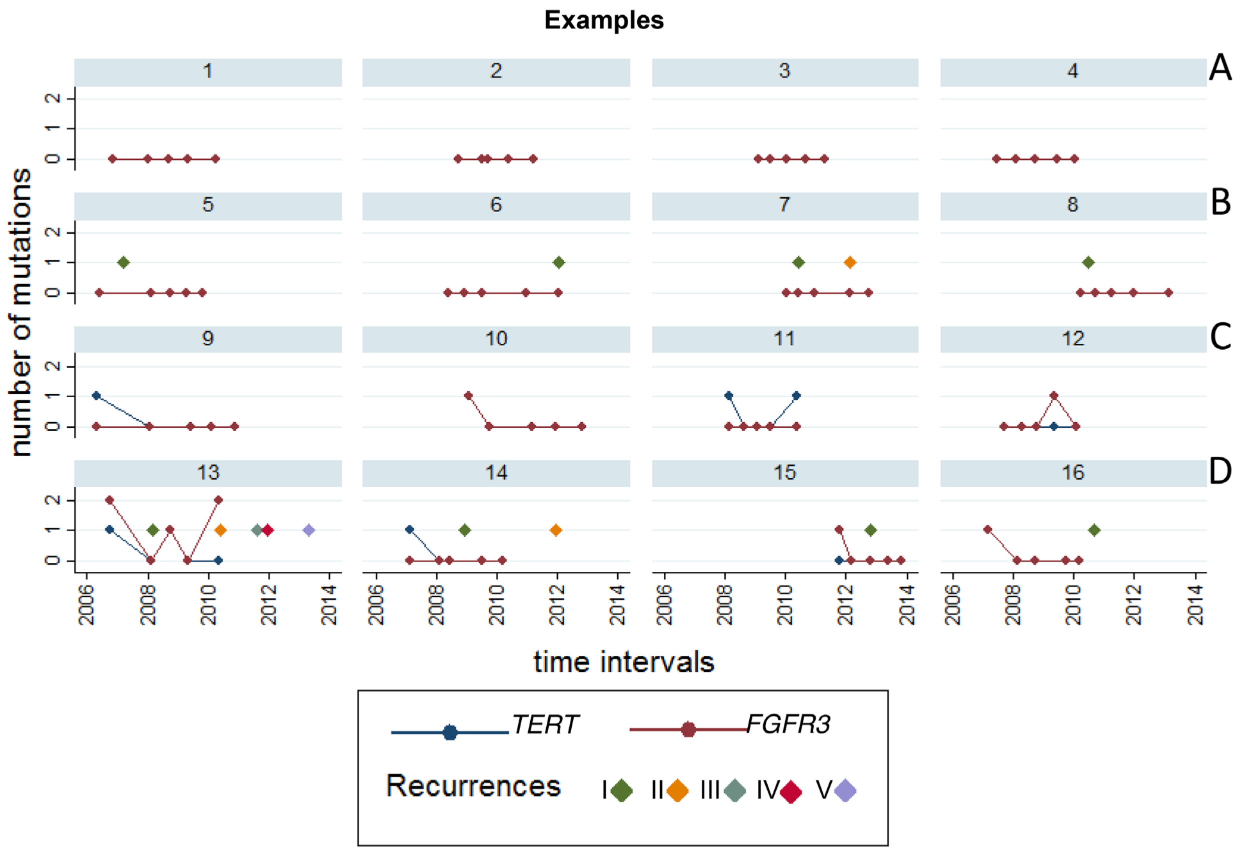

Supplementary Figure S2: Examples showing the variegated scenario of mutations in *TERT* and *FGFR3* and event(s) of recurrences in the subgroup of NMIBC patients repeatedly followed overtime. Next to each category the frequency of subjects with mutations is reported: **A.** patients with no mutations and no recurrences during follow-up (true negative); **B.** patients with recurrences during follow-up but in whose urinary exfoliated cells no mutations were detected after diagnosis (false negative); **C.** patients who did not recurred over time but with mutations (false positive) and, finally, **D.** patients whose samples showed mutation(s) and recurred over time (true positive).

Supplementary Table S1: Distributions of mutations in urinary exfoliated cells of investigated genes according to clinical and demographic characteristics of MIBC patients

See Supplementary File 1

Supplementary Table S2: Overall and cancer survival for all subjects (NMIBC and MIBC, n=255) of the study in relation to investigated gene mutations at diagnosis

Genes	Overall survival		Cancer survival	
	OR (95% CI)	p-value	OR (95% CI)	p-value
**All genes combined**				
1 gene mutated	3.43 (1.10-10.69)	**0.03**	2.55 (0.64-10.23)	0.19
2 or more	3.51(1.05-11.69)	**0.04**	2.64 (0.60-11.52)	0.20
TERT+FGFR3+PIK3CA				
1 gene mutated	3.48 (1.12-10.81)	**0.03**	2.59 (0.65-10.35)	0.18
2 or more	3.75 (1.13-12.50)	**0.03**	2.81 (0.64-12.28)	0.17
TERT+FGFR3+Ras				
1 gene mutated	3.55 (1.14-11.06)	**0.03**	2.61 (0.65-10.47)	0.18
2 or more	3.97 (1.19-13.24)	**0.03**	2.96 (0.67-12.96)	0.15
TERT+FGFR3				
1 gene mutated	3.53 (1.14-10.97)	**0.03**	2.61 (0.65-10.44)	0.18
2 or more	4.43 (1.32-14.81)	**0.02**	3.25 (0.74-14.25)	0.12
TERT	2.22 (0.94-5.24)	0.07	2.54 (0.83-7.78)	0.10
FGFR3	2.43 (1.04-5.70)	**0.04**	1.55 (0.56-4.33)	0.40
PIK3CA	0.53 (0.11-2.51)	0.42	1.11 (0.22-5.68)	0.89
Ras	2.65 (0.58-12.12)	0.21	2.97 (0.52-16.84)	0.22

Adjusted for age, smoking status, age, grade, stage and therapy. Significant results in bold.

Supplementary Table S3: Event of recurrence or number of recurrences in NMIBC (n=230) in associations with mutations in analyzed genes, in different combinations or individually, at diagnosis

Genes	Recurrence yes/no		Number of recurrences	
	OR (95% CI)	p-value	OR (95% CI)	p-value
**All genes combined**				
1 gene mutated	1.71 (0.82- 3.54)	0.15	1.85 (0.91-3.75)	0.01
2 or more	1.83 (0.92-3.66)	0.09	2.05 (1.05-3.98)	**0.04**
TERT+FGFR3+PIK3CA				
1 gene mutated	1.82 (0.89-3.76)	0.10	1.95 (0.97-3.93)	0.06
2 or more	2.01 (1.0-4.06)	**0.05**	2.25 (1.15-4.41)	**0.02**
TERT+FGFR3+Ras				
1 gene mutated	1.94 (0.95-3.98)	0.07	2.02 (1.01-4.04)	**0.04**
2 or more	1.88 (0.93-3.79)	0.08	2.16 (1.10-4.25)	**0.03**
TERT+FGFR3				
1 gene mutated	1.97 (0.97-4.01)	0.06	2.04 (1.03-4.05)	**0.04**
2 or more	2.18 (1.06-4.46)	**0.03**	2.51 (1.26-5.00)	**0.01**
TERT	1.83 (1.01-3.34)	**0.04**	1.95 (1.10-3.44)	**0.02**
FGFR3	1.57 (0.89-2.78)	0.12	1.76 (1.02-3.06)	**0.04**
PIK3CA	1.24 (0.56-2.77)	0.59	1.22 (0.57-2.61)	0.60
Ras	0.14 (0.017-1.12)	0.06	0.14 (0.02-1.11)	0.06

Adjusted for age, smoking status, age, grade, stage and therapy. Significant results in bold.
