# Supplementary material for: Detection of multiple mutations in urinary exfoliated cells from male bladder cancer patients at diagnosis and during follow-up
Source: Oncotarget. 2016 Sep 7;7(41):67435–48. doi: 10.18632/oncotarget.11883 (PMC5341887; doi:10.18632/oncotarget.11883)
Supplement: Supplementary file 2 [file oncotarget-07-67435-s002.docx]

**Supplementary Table 1.** Distributions of mutations in urinary exfoliated cells of investigated genes according to clinical and demographic characteristics of MIBC patients.

|  | ***TERT*** | | | ***FGFR3*** | | ***PIK3CA*** | | ***Ras*** | |
| --- | --- | --- | --- | --- | --- | --- | --- | --- | --- |
| **Variables** | **No Mutations N (%)** | **≥1 Mutations N (%)** | | **No Mutations N (%)** | **≥1 Mutations N (%)** | **No Mutations N (%)** | **≥1 Mutations**  **N (%)** | **No Mutations N (%)** | **≥1 Mutations**  **N (%)** |
|  |  |  |  |  |  |  |  |  |  |
| **All patients** | 5 (20.0) | 20 (80.0) | | 20 (83.3) | 4 (16.7) | 24 (96.0) | 1 (4.0) | 25 (100) | 0 (0.0) |
| **Age** (years) Mean ± SD | 66.9 ± 5.9 | 64.2± 9.2 | | 65.1±7.4 | 62.5±15.2 | 65.7±7.0 | 40.2 | 64.7±8.6 |  |
|  | p= 0.54 | | | p= 0.60 | | - | | - | |
| **Smoking status** |  | |  |  |  |  |  |  |  |
| Never | 2 (40.0%) | | 11 (55.0%) | 2 (10.0%) | 1 (25.0%) | 3 (12.5%) | 0 | 3 (12.0%) | 0 |
| Former | 2 (40.0%) | | 7 (35.0%) | 8 (40.0%) | 1 (25.0%) | 9 (37.5%) | 0 | 9 (36.0%) | 0 |
| Current | 1 (20.0%) | | 2 (10.0%) | 10 (50.0%) | 2 (50.0%) | 12 (50.0%) | 1 (100.0%) | 13 (52.0%) | 0 |
|  | p= 0.77 | | | p= 0.67 | | p= 0.62 | |  | |
| **Grading (1973)** |  | |  |  |  |  |  |  |  |
| G2 | 0 | | 1 (5.0%) | 0 | 1 (25.0%) | 1 (4.2%) | 0 | 1 (4.0%) | 0 |
| G3 | 5 (100.0%) | | 19 (95.0%) | 20 (100.0%) | 3 (75.0%) | 23 (95.8%) | 1 (100.0%) | 24 (96.0%) | 0 |
|  | p= 0.61 | | | **p= 0.02** | | p= 0.83 | |  | |
| **Tumor size** |  | |  |  |  |  |  |  |  |
| <3cm | 1 (20.0%) | | 6 (30.0%) | 4 (20.0%) | 2 (50.0%) | 7 (29.2%) | 0 | 7 (28.0%) | 0 |
| ≥3cm | 4 (80.0%) | | 14 (70.0%) | 16 (80.0%) | 2 (50.0%) | 17 (70.8%) | 1 (100.0%) | 18 (72.0%) | 0 |
|  | p= 0.65 | | | p= 0.20 | | p= 0.52 | |  | |
| **Stage** |  | |  |  |  |  |  |  |  |
| T2 | 3 (60.0%) | | 20 (100.0%) | 18 (90.0%) | 4 (100.0%) | 22 (91.6%) | 1 (100.0%) | 23 (92.0%) | 0 |
| T3 | 1 (20.0%) | | 0 | 1 (5.0%) | 0 | 1 (4.2%) | 0 | 1 (4.0%) | 0 |
| T4 | 1 (20.0%) | | 0 | 1 (5.0%) | 0 | 1 (4.2%) | 0 | 1 (4.0%) | 0 |
|  | **p= 0.01** | | | p= 0.80 | | p= 0.96 | |  | |
| **Progression to extravesical disease** |  | |  |  |  |  |  |  |  |
| No | 3 (60.0%) | | 12 (60.0%) | 13 (65%) | 1 (25.0%) | 15 (62.5%) | 0 | 15 (60.0%) | 0 |
| Yes | 2 (40.0%) | | 8 (40.0%) | 7 (35.0%) | 3 (75.0%) | 9 (37.5%) | 1 (100.0%) | 10 (40.0%) | 0 |
|  | p= 1.00 | | | p= 0.14 | | p= 0.21 | |  | |
| **Type of progression** |  | |  |  |  |  |  |  |  |
| Local | 1 (50.0%) | | 2 (25.0%) | 2 (28.6%) | 1 (33.3%) | 3 (33.3%) | 0 | 3 (30.0%) | 0 |
| Distal | 0 | | 1 (12.5%) | 0 | 1 (33.3%) | 1 (11.1%) | 0 | 1 (10.0%) | 0 |
| Local+Distal | 1 (50.0%) | | 5 (62.5%) | 5 (71.4%) | 1 (33.3%) | 5 (55.6%) | 1 (100.0%) | 6 (60.0%) | 0 |
|  | p= 1.00 | | | p= 0.46 | | p= 1.00 | | - | |
| **Survival at follow-up** |  | |  |  |  |  |  |  |  |
| Alive (or died due to other causes other than BC) | 4 (80.0%) | | 12 (60.0%) | 14 (70.0%) | 2 (50.0%) | 16 (66.6%) | 0 | 16 (64.0%) | 0 |
| Dead | 1 (20.0%) | | 8 (40.0%) | 6 (30.0%) | 2 (50.0% | 8 (33.4%) | 1 (100.0%) | 9 (36.0%) | 0 |
|  | p= 0.41 | | | p= 0.44 | | p= 0.17 | | p= 0.49 | |

Significant results in bold.
